# Predictive factors associated with bile culture positivity: a model development and diagnostic test accuracy study

**DOI:** 10.1007/s00464-025-12291-2

**Published:** 2025-11-11

**Authors:** Camilo Ramírez-Giraldo, Carlos Rodriguez Barbosa, Violeta Avendaño-Morales, Samir Moreno-Martínez, Isabella Van-Londoño, Susana Rojas-López, Andrés Isaza-Restrepo

**Affiliations:** 1 Hospital Universitario Mayor - Méderi, Bogotá, Colombia; 2 Grupo de Investigación Clínica, Universidad del Rosario, Bogotá, Colombia

**Keywords:** Antibiotics, Cholecystitis, Cholecystectomy, Clinical decision rules

## Abstract

**Background:**

The role of antibiotics in the preoperative and perioperative management of patients with benign biliary pathology is still not clear. This study aims to develop a predictive model for identifying patients with benign biliary disease who are at low risk of positive bile cultures, optimizing antibiotic administration.

**Methods:**

Prospective cohort study conducted between April and October 2024 in a single center from a hospital network with two locations. Consecutive adult patients (aged ≥ 18 years) diagnosed with benign biliary disease who underwent cholecystectomy with intraoperative bile culture sampling. The primary validation outcome was positive bile cultures; predictors included demographic, clinical, and paraclinical variables.

**Results:**

Overall, 703 patients were included in the study. The incidence of positive cultures was 32.1%. The rate of major complications (Clavien-Dindo ≥ 3), comorbidities, age, and higher cholecystitis severity was significantly higher in patients with positive bile cultures. The multivariable prediction model for positive cultures included age, ERCP, and time from admission to procedure, showing fair discrimination (c-statistic 0.75, 95% CI 0.70–0.80) with no evidence of poor calibration. Patients classified as low risk for positive bile cultures had a sensitivity of 92% and a negative likelihood ratio of 0.21, indicating that classification in the low-risk group effectively rules out the possibility of a positive bile culture.

**Conclusion:**

This study found our predictive model useful in assessing bile culture positivity during cholecystectomy to rule out the need for antibiotic initiation in low-risk patients, both preoperatively while awaiting surgery (in cases of acute cholecystitis) and perioperatively (prophylaxis).

**Supplementary Information:**

The online version contains supplementary material available at 10.1007/s00464-025-12291-2.

Antibiotic therapy is commonly used alongside laparoscopic cholecystectomy, including preoperative antibiotics (administered from admission until surgery in cases of cholecystitis). Suggested schemes consider perioperative prophylaxis (administered within 120 min before the procedure) and, in selected cases, postoperative antibiotics [1].

Pre-operative antibiotics are empirically administered in cases of acute cholecystitis, following current guidelines, despite the lack of robust evidence on their utility [2, 3]. Moreover, this practice is not consistent with current understanding of the pathophysiology of acute cholecystitis, where inflammation results primarily from a mechanical effect, with a secondary infectious process occurring in some cases [4, 5]. Nonetheless, infection is neither a necessary nor a sufficient cause for the presentation of acute cholecystitis [1, 6].

On the other hand, the role of perioperative antibiotics in reducing postoperative complications remains a subject of debate. Some evidence suggests that omitting antibiotic therapy in patients with positive bile cultures may increase the risk of postoperative infectious complications [7].

Most studies have adopted a generalized approach, comparing the strategy of routine antibiotic administration in all patients versus omitting antibiotics entirely. Results suggest that indiscriminate antibiotic administration in all patients leads to unnecessary antibiotic use, increasing healthcare costs, adverse events, and antibiotic resistance [8].

Given these concerns, it seems important to identify patients with a low likelihood of having positive bile cultures to avoid unnecessary preoperative and perioperative antibiotic administration.

This study aims to develop a predictive model for identifying patients with benign biliary disease who are at low risk of positive bile cultures, optimizing antibiotic administration.

## Methods

### Study design and participants

A prospective cohort study was designed for the development and validation of a clinical prediction model at a single center comprising of two locations from the same hospital network. The study protocol was published beforehand (Predictive factors associated with Bile culture positivity And phenotypiCal antIbiogram resistance patterns in patients taken to LaparOscopic cholecystectomy (BACILO)) [9]. All patients admitted to the institution between April 2024 and October 2024 who met eligibility criteria were included in the study. Data for all variables were collected in an anonymous database using REDCap electronic data capture tools [10]. This study was reviewed and approved by our institution’s ethics committee (approval number 2555-CV1837). We obtained written informed consent from all participants. The study adhered to the TRIPOD + AI statement: updated guidance for reporting clinical prediction models that use regression or machine learning methods (Supplement 1) [11].

We included consecutive adult patients (aged ≥ 18 years) diagnosed with benign biliary disease who underwent cholecystectomy with intraoperative bile culture sampling. In cases of cholecystitis or cholangitis, empirical antibiotic therapy was initiated in accordance with the Tokyo Guidelines and continued until the time of surgery [2]. Additionally, all patients received prophylactic antibiotics within 120 min prior to skin incision, following institutional protocols. Patients were excluded if they underwent laparoscopic cholecystectomy combined with another concomitant surgical procedure, did not have a postoperative follow-up appointment, or had a previously documented diagnosis of gallbladder or biliary tract malignancy.

### Predictive factors

Assessed predictive factors included demographic, clinical, and laboratory characteristics. Predictors were identified based on a prior literature review and were selected if they were both plausible and statistically significant in the bivariate analysis.

### Reference standard

The bile culture result was the reference standard. Bile was collected intraoperatively from the surgical specimen, anonymously labeled, and sent for analysis in our institutions’ laboratory. The bile sample was obtained by skin puncture using a #14 French catheter directly into the gallbladder wall. Bile samples were inoculated on agar plates, including MacConkey, thioglycolate, and both microaerophilic and GasPak anaerobic chocolate agar plates. They were then incubated for 24, 48, and 72 h in an aerobic environment and up to 240 h in an anaerobic environment. For positive cultures, the antibiogram was processed using the Phoenix M50 system, and carbapenem resistance was detected through polymerase chain reaction (PCR). If any yeast growth was detected, the agar was sent to an index laboratory, where it was processed using a microdilution technique [9].

### Sample size calculation

The sample size calculation was based on a proportion of positive cultures of 52.12% and an antibiotic resistance rate of 25.25%. The sample size was determined using statistical formulas for prognostic prediction models [12]. This was calculated using Stata V.18. A total of seven predictors were considered (age, diabetes, previous ERCP, and diagnosis and severity of cholecystitis according to Tokyo criteria, C-reactive protein) from a review of the literature. The final estimated sample size for the outcome of positive bile culture was 636 patients.

### Statistical analysis

A description of demographic, clinical, paraclinical, intraoperative, and surgical outcome variables was performed. Categorical variables were described as proportions, ﻿whereas continuous variables were described as means and standard deviations (SD). A bivariate analysis was performed using the χ^2^ test (or Fisher’s exact test, when appropriate) for categorical variables and the two-tailed t test for continuous variables to compare differences based on whether the bile culture was positive or negative.

Binary logistic regression models were fitted using a manual stepwise backward elimination approach, sequentially removing variables based on both statistical significance (p value < 0.05) and clinical plausibility. The results of the final multivariable model are presented with their coefficients (Beta) and odds ratios (OR) along with their corresponding 95% confidence intervals (95% CI). Multicollinearity and linearity were assessed using the variance inflation factor and the Box–Tidwell test, respectively.

We evaluated the overall model performance using the Brier score and the Akaike Information Criterion.

### Validation and assessment of the model

We evaluated model discrimination with the concordance (c) statistic and discrimination slope and displayed the discrimination graphically using a receiver operating characteristic plot; the optimal cutoff point with the highest diagnostic performance was determined using the Youden Index method, and we calculated its sensitivity, specificity, and likelihood ratios (LR + and LR-). We assessed model calibration with the Hosmer–Lemeshow goodness-of-fit test and calibration plots. We used 500 random bootstrap samples with replacement from the full sample of participants, constructed models on these bootstrap samples, and derived optimism-adjusted performance measures to provide a realistic estimate of future performance. We conducted a sensitivity analysis by excluding outliers from the model, assessing its performance, and comparing the results with the full dataset. The net benefit was derived by calculating the difference between the true positive rate and weighed false positive rate across different threshold probabilities in the cohort. The decision curve was plotted against the threshold probability.

All analyses were carried out using Stata® V.18 and RStudio® (2023.12.1 + 402).

## Results

**Fig. 1:**
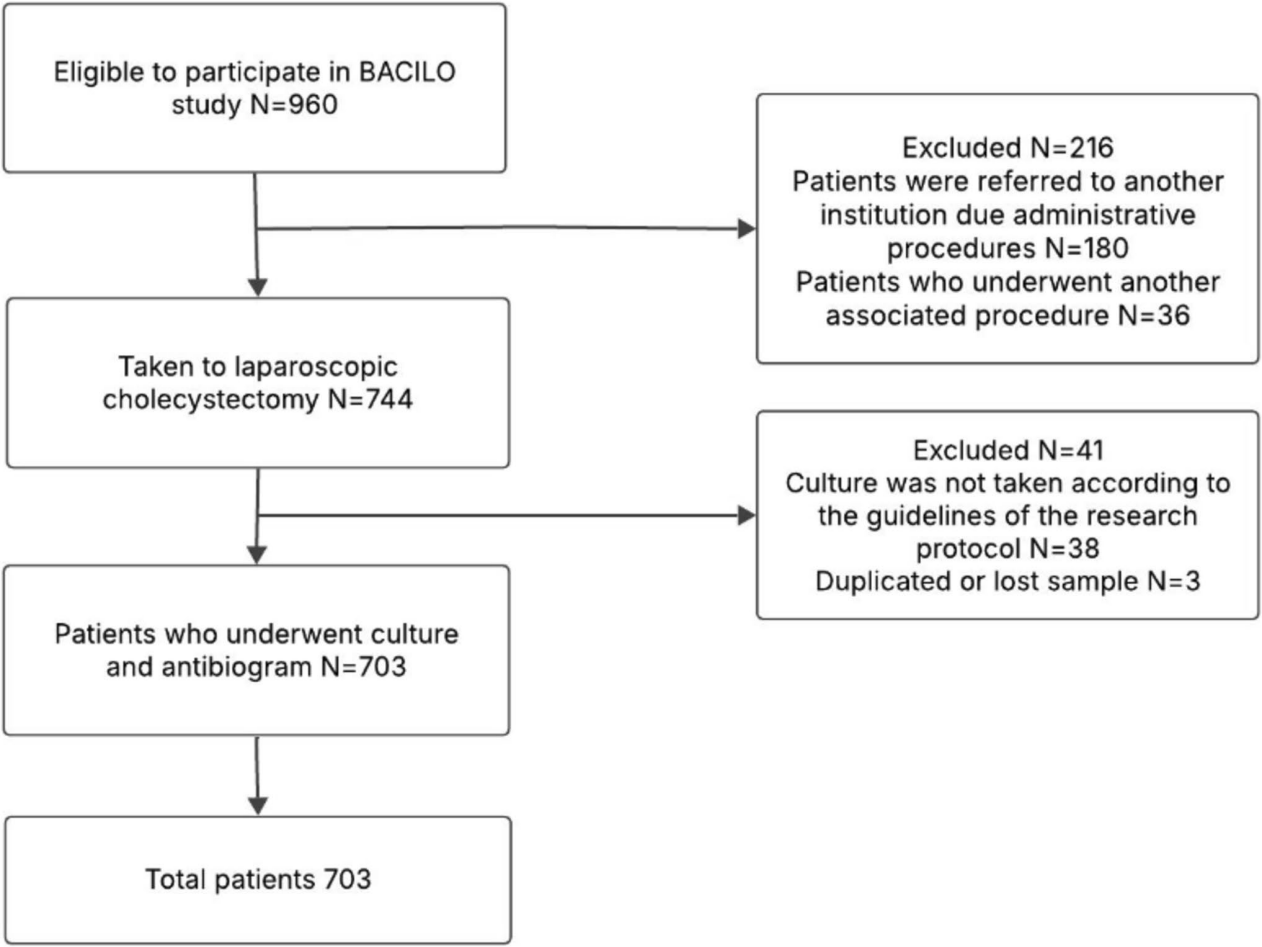
Flowchart selection process

A total of 703 patients were included in the study, with a flowchart showing the selection process (Fig. 1). The incidence of positive cultures was 32.1%. The mean age of patients was 53.8 ± 19.2 years, with the majority being female (65.6%). The most common indication for cholecystectomy was cholecystitis (52.2%). When comparing characteristics based on bile culture results, we found that age was higher in patients with positive cultures. Similarly, the proportion of patients with diabetes, hypertension, and cardiovascular disease was greater among those with positive cultures. Regarding severity based on the Tokyo classification, a higher proportion of positive cultures was observed in patients with Tokyo III cholecystitis. Further demographic, clinical, and surgical characteristics are detailed in Table 1.

**Table 1:** Demographic, clinical, paraclinical and intraoperative characteristics based on whether the bile culture was positive or negative

	N (%)	Negative culture (*n = *477)	Positive cultures (*n = *226)	p value
Age (mean)(SD)(years)	53.8 ± 19.2	48.9 ± 18.3	63.9 ± 16.7	< 0.001*
Sex				1.000
Female	461 (65.6)	313 (65.6)	148 (65.5)	
Male	242 (34.4)	164 (34.4)	78 (34.4)	
Body mass index (mean)(SD)(kg/m^2^)	27.0 ± 4.6	27.1 ± 4.8	26.9 ± 4.3	0.451*
ASA				** < 0.001**
I	338 (48.1)	272 (57.0)	66 (29.2)	
II	237 (33.7)	143 (30.0)	94 (41.6)	
III	113 (16.1)	57 (12.0)	56 (24.8)	
IV	15 (2.1)	5 (1.0)	10 (4.4)	
Co-morbidity				
Arterial hypertension	222 (31.6)	114 (23.9)	108 (47.9)	** < 0.001**
Diabetes mellitus	90 (12.8)	41 (8.6)	49 (21.7)	** < 0.001**
Chronic obstructive pulmonary disease	39 (5.5)	11 (2.3)	28 (12.4)	** < 0.001**
Chronic kidney disease	22 (3.1)	14 (2.9)	8 (3.5)	0.843
Cardiovascular disease	54 (7.7)	22 (4.6)	32 (14.2)	** < 0.001**
Liver disease	6 (0.9)	6 (1.3)	0 (0.0)	0.210
Charlson comorbidity index (mean) (points)	1.8 ± 2.0	1.3 ± 1.8	2.8 ± 2.1	< 0.001*
Pre-operative laboratories (mean)(SD)				
Leukocytes (× 10^3^)	10.6 ± 4.4	10.5 ± 4.3	10.8 ± 4.6	0.429*
Hemoglobin (mg/dL)	14.6 ± 1.8	14.6 ± 1.8	14.4 ± 1.9	0.280*
Bilirubin (mg/dL)	1.6 ± 2.3	1.4 ± 1.9	2.0 ± 2.8	0.001*
Alkaline phosphatase (mg/dL)	157.4 ± 146.4	141.3 ± 117.6	191.3 ± 189.8	< 0.001*
Aspartate aminotransferase (mg/dL)	110.4 ± 189.8	98.8 ± 165.3	135.3 ± 232.3	0.018*
Alanine aminotransferase (mg/dL)	126.4 ± 206.2	115.1 ± 190.8	150.6 ± 234.2	0.034*
C-reactive protein (mg/dL)	6.2 ± 9.2	5.1 ± 8.7	8.4 ± 9.9	0.005*
Imaging findings				
Bile duct diameter (mean)(SD)(mm)	5.0 ± 2.2	4.6 ± 1.8	5.5 ± 2.7	< 0.001*
Gallbladder wall thickness (mean)(SD)(mm)	3.9 ± 1.8	3.8 ± 1.9	3.7 ± 1.3	0.434*
Scleroatrophic gallbladder	6 (0.9)	5 (1.0)	1 (0.4)	0.707
Perforation	13 (1.8)	4 (0.8)	9 (4.0)	**0.010**
Tokyo severity				**0.001**
Without cholecystitis	336 (47.8)	232 (48.6)	104 (46.0)	
I	144 (20.5)	106 (22.2)	38 (16.8)	
II	154 (21.9)	107 (22.4)	47 (20.8)	
III	69 (9.8)	32 (6.8)	37 (16.4)	
Pre-operative ERCP				** < 0.001**
No	589 (83.8)	426 (89.3)	163 (72.1)	
Yes	114 (16.2)	51 (10.7)	63 (27.9)	
History of cholecystostomy				0.482
No	691 (98.3)	469 (98.3)	222 (98.2)	
Yes	12 (1.7)	8 (1.7)	4 (1.8)	
Intraoperative findings according to Nassar score				** < 0.001**
1	172 (24.5)	132 (27.7)	40 (17.7)	
2	231 (32.9)	163 (34.2)	68 (30.1)	
3	139 (19.8)	96 (20.0)	43 (19.0)	
4	83 (11.8)	48 (10.1)	35 (15.5)	
5	78 (11.0)	38 (8.0)	40 (17.7)	
Time from admission to procedure (mean)(SD)(days)	4.1 ± 5.4	3.3 ± 4.3	5.7 ± 6.8	< 0.001*

p values were obtained using the chi-squared test

^*^p values were obtained using the 2-tailed t test

Bold values indicate statistically significant p values (p < 0.05)

﻿﻿In terms of surgical outcomes, a higher proportion of major complications (Clavien-Dindo ≥ 3) was observed in patients with positive cultures, which was yielded as statistically significant. Similarly, the proportion of bailout procedures, drain use, surgical site infection, mortality, and surgical time was higher in the positive culture group, although these differences were not statistically significant (Table 2).

**Table 2:** Surgical outcomes based on whether the bile culture was positive or negative

	N (%)	Negative culture (*n = *477)	Positive cultures (*n = *226)	p value
Conversion to open				0.482
No	694 (98.9)	472 (99.2)	222 (98.2)	
Yes	8 (1.1)	4 (0.8)	4 (1.8)	
Type of cholecystectomy				0.241
Total	665 (94.6)	455 (95.4)	210 (92.9)	
Subtotal	38 (5.4)	22 (4.6)	16 (7.1)	
Surgical site infection				0.083
No	689 (98.0)	471 (98.7)	218 (96.5)	
Yes	14 (2.0)	6 (1.3)	8 (3.5)	
Drain use				0.132
No	654 (93.0)	449 (94.1)	205 (90.7)	
Yes	49 (7.0)	28 (5.9)	21 (9.3)	
Surgical time (median)(IQR)(minutes)	83.2 ± 40.8	81.3 ± 38.9	87.0 ± 44.2	0.088*
Postoperative hospital stay (median)(IQR)(days)	1.7 ± 5.5	1.5 ± 4.5	2.2 ± 7.0	0.089*
Major complication (Clavien-Dindo ≥ 3)				**0.001**
No	683 (97.2)	471 (98.7)	212 (93.8)	
Yes	20 (2.8)	6 (1.3)	14 (6.2)	
30-day mortality				0.168
No	697 (99.1)	475 (99.6)	222 (98.2)	
Yes	6 (0.9)	2 (0.4)	4 (1.8)	

p values were obtained using the chi-squared test

^*^p values were obtained using the 2-tailed t test

Bold values indicate statistically significant p values (p < 0.05)

The multivariable prediction model for positive bile cultures included three predictors: age, endoscopic retrograde cholangiopancreatography (ERCP), and time from admission to procedure (Table 3). This model was conducted without imputed data. The model calculates the predicted probability of positive bile culture (the predictor values for ERCP are 1 when present and 0 when absent):

$$\ Predicted\,probability = \frac{1}{{1 + \exp^{ - BACILO\,risk\,score} }}$$

$$BACILO risk score = - 3.469 + 0.042 \left( {age} \right) + 0.832 \left( {ERCP} \right) + 0.036 \left( {time from admision to procedure} \right)$$

**Table 3:** Multivariable logistic regression prediction model for positive bile cultures

	β coefficient (SE)	Odds ratio (95% CI)	p value
Intercept	− 3.469 (0.31)		** < 0.001**
Age (years)	0.042 (0.00)	1.04 (1.03–1.05)	** < 0.001**
ERCP	0.832 (0.23)	2.30 (1.44–3.68)	** < 0.001**
Time from admission to procedure (days)	0.036 (0.01)	1.04 (1.00–1.07)	**0.026**

Bold values indicate statistically significant p values (p < 0.05)

All three predictors were independently associated with positive bile cultures. The variance inflation factor values (age 1.01, ERCP 1.09, and time from admission to procedure 1.10) confirmed no evidence of multicollinearity between predictors, and the Box–Tidwell test confirmed the assumption of linearity. We did a sensitivity analysis excluding outliers, which showed similar significance, direction, and magnitude of the independent associations.

**Fig. 2:**
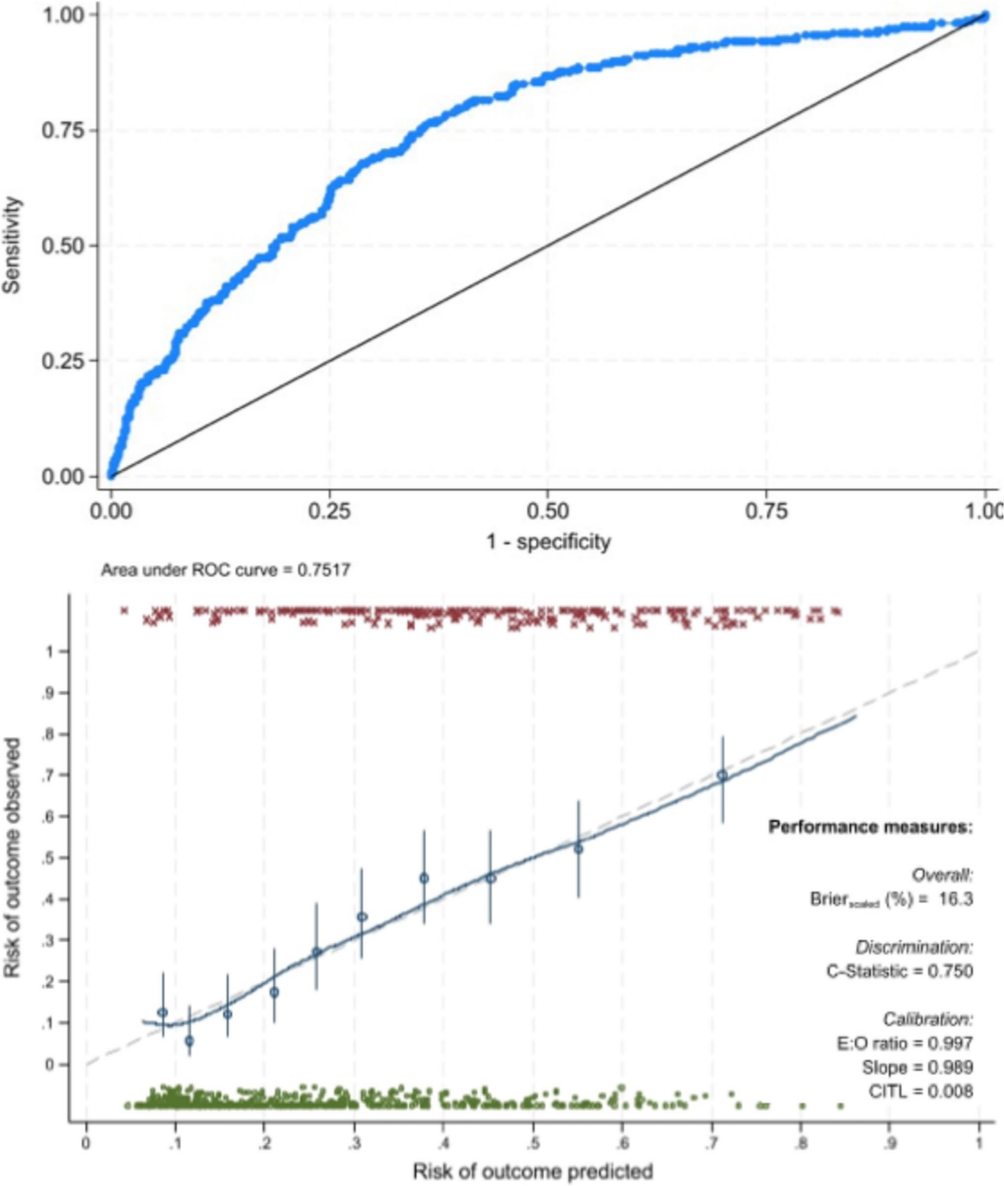
Discrimination and calibration measures of prediction model performance

The model showed fair discrimination (c-statistic 0.75, 95% CI 0.70–0.80) with no evidence of poor calibration (Hosmer–Lemeshow goodness-of-fit test *p = *0.417). Internal validation identified small differences in the overall performance measures, with no evidence of poor calibration (Fig. 2).

**Fig. 3:**
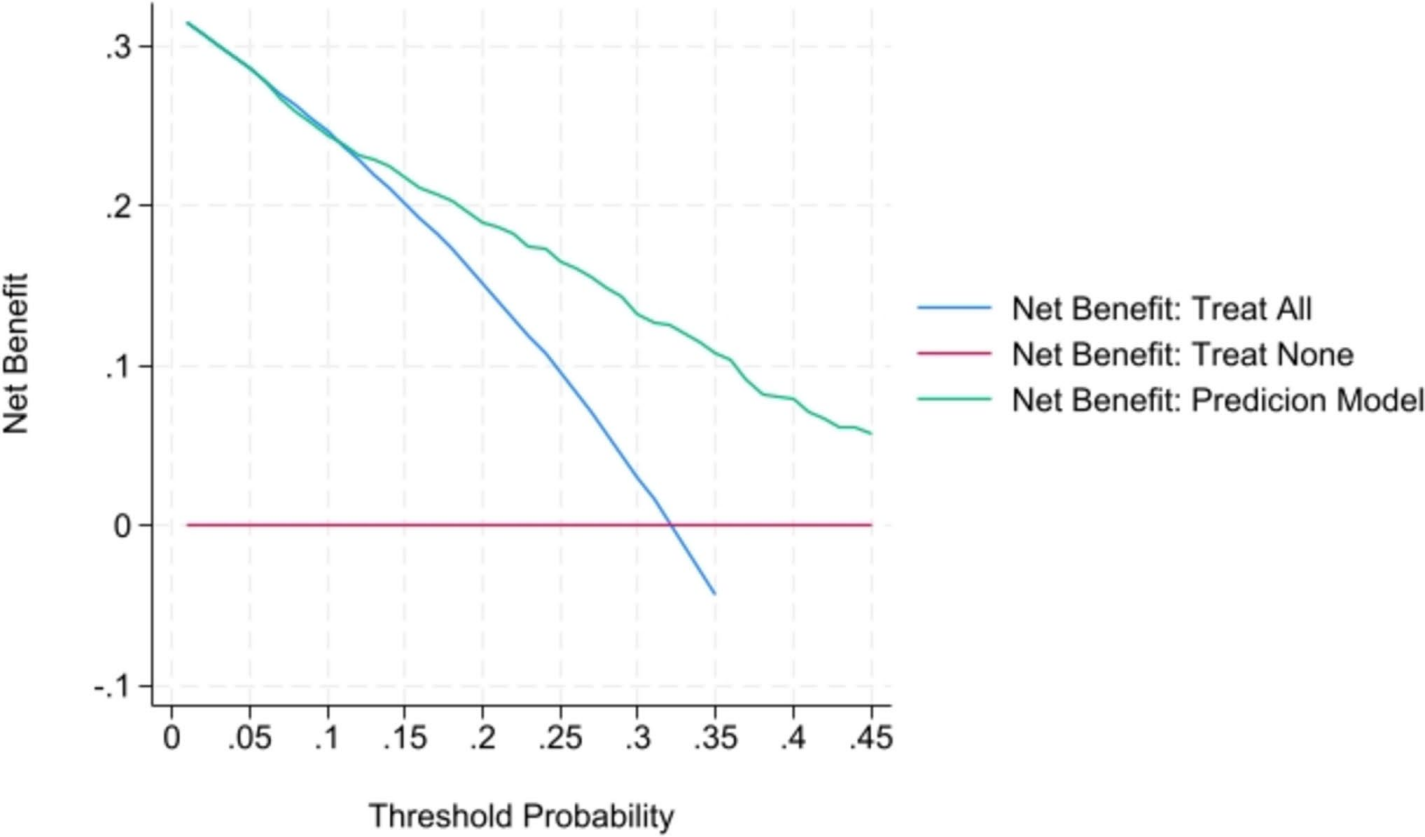
Decision curve analysis

The cutoff point with the highest diagnostic performance, according to the Youden Index, was −0.99, with a sensitivity of 0.78, specificity of 0.61, a LR + of 2.05, and a LR- of 0.34 for predicting positive bile cultures. Decision curve analysis showed that the BACILO model gained more net benefits than the treat-all patients strategy and the treat-none strategy (Fig. 3).

Patients in approximately the upper (≥ 75th percentile) and lower (≤ 25th percentile) quartiles of the BACILO risk score distribution were classified as high and low risk, respectively. Specifically, patients with a risk score lower than −1.71 (*n = *176) were categorized as low risk (sensitivity: 0.92, specificity: 0.33, LR + : 1.39, LR-: 0.21); patients with a risk score higher than −0.26 (*n = *178) were classified as high risk (sensitivity: 0.45, specificity: 0.84, LR + : 2.83, LR-: 0.65); and patients with intermediate scores between these thresholds (*n = *349) were designated as medium risk. Based on this classification, the proportion of patients with positive cultures was 9.1% (*n = *16), 30.9% (*n = *108), and 57.3% (*n = *102) for the low-, medium-, and high-risk groups, respectively. Supplement 2 provides further details on demographic, clinical, paraclinical, intraoperative characteristics, and surgical outcomes according to risk classification.

Another predictive variable included in the model was C-reactive protein (CRP), which has been previously described in the literature and showed statistically significant differences between patients with positive and negative bile cultures (*p = *0.005). However, CRP was not incorporated into the original model due to a high proportion of missing data (58.2%). We evaluated the diagnostic accuracy of a model incorporating CRP. This model demonstrated fair discrimination (c-statistic: 0.76, 95% CI: 0.70–0.80) with no evidence of poor calibration (Hosmer–Lemeshow goodness-of-fit test, *p = *0.063). The inclusion of CRP improved model performance, as indicated by a higher model fit (model without CRP: χ^2^ = 8.16, Akaike information criterio*n = *766.7 vs. model with CRP: χ^2^ = 14.79, Akaike information criterion = 316.5).

## Discussion

In our study, we identified a three-variable model incorporating age, ERCP, and time from admission to surgery to predict positive bile cultures. The model demonstrated fair discrimination and excellent calibration. We identified a clinically meaningful diagnostic cutoff with potential implications for future clinical practice. Specifically, patients classified as low risk for positive bile cultures had a sensitivity of 92% and a negative likelihood ratio of 0.21, indicating that classification in the low-risk group effectively rules out the possibility of a positive bile culture.

When comparing patients with a low risk of positive bile cultures to those at intermediate or high risk, we observed that low-risk patients had a significantly lower proportion of bailout procedures, reduced drain use, a lower frequency of difficult cholecystectomies (according to intraoperative findings based on the Nassar score for difficult cholecystectomy), longer surgical times, and a higher proportion of major complications. This suggests that the factors identified as predictors of positive bile cultures may also be associated with difficult cholecystectomy. It is worth noting that among patients with severe acute cholecystitis, only 1.7% were classified as low risk in the model.

The predictive factors identified in our study have previously been described in the literature as associated with positive bile cultures, whereas others—such as ASA classification, comorbidities, and the presence of cholecystitis—were not found to be predictive in our model [13–16].

The available evidence on the use of preoperative antibiotics in this setting is limited. In a recent systematic review conducted by our group [1], we identified two randomized controlled trials evaluating antibiotic use in this context, neither of which found statistically significant differences in complication rates. Notably, these studies included patients with mild or moderate cholecystitis without local complications [17, 18].

Regarding perioperative antibiotic use, a systematic review concluded that there is no significant difference in outcomes between administering and withholding antibiotics in cases of mild to moderate cholecystitis [19]. However, another systematic review that included low-risk patients undergoing elective cholecystectomy found that antibiotics reduced the incidence of infectious complications [20]. Furthermore, a clinical trial reported that omitting antibiotic administration in patients with positive bile cultures was a risk factor for surgical site infection [7].

Given the limited and contradictory evidence, we propose that a selective approach to antibiotic administration is the most appropriate strategy. Antibiotics should be withheld in cases with a low risk of positive bile cultures.

These diagnostic criteria could guide decisions regarding preoperative and perioperative antibiotic therapy. It is essential to emphasize that this risk score serves as a decision-support tool for antibiotic use initiation but does not replace clinical judgment.

This study has several strengths. We minimized selection bias by employing a prospective design and including all eligible patients in the model. Additionally, we achieved the target sample size and conducted internal validation using bootstrapping to reduce optimism in performance estimates.

Nonetheless, this study has several limitations**.** Its single-center design may limit generalizability, and we encountered missing data for certain variables, such as C-reactive protein (CRP), which could have added predictive value. Additionally, immunosuppression was not accounted for, which may introduce some residual confounding.

We are currently planning an external validation study to evaluate the performance and generalizability of the prediction model and diagnostic criteria across diverse clinical settings, countries, ethnic backgrounds, and patient populations. This will also enable us to assess the reproducibility of predictor measurement by independent investigators. In parallel, we are designing a follow-up study that incorporates C-reactive protein (CRP) to investigate whether its inclusion improves the model’s discriminatory accuracy.

## Conclusion

We propose that this model could be used to rule out the need for antibiotic initiation in low-risk patients, both preoperatively while awaiting surgery (in cases of acute cholecystitis) and perioperatively.

## Supplementary Information

Below is the link to the electronic supplementary material.

Supplementary material 1 (PDF 576.9 kb)

Supplementary material 2 (PDF 72.4 kb)

## Data Availability

Demographic, clinical, paraclinical, intraoperative, and surgical outcome data used in this study are available online (Dataverse), along with the code for logistic regression and internal validation. 10.34848/PYATTI
