# Supplementary material for: Predictive factors associated with bile culture positivity: a model development and diagnostic test accuracy study
Source: Surg Endosc. 2025 Nov 11;40(2):1157–66. doi: 10.1007/s00464-025-12291-2 (PMC12881028; doi:10.1007/s00464-025-12291-2)
Supplement: Supplementary file 2 — Supplementary material 2 (PDF 72.4 kb) [file 464_2025_12291_MOESM2_ESM.pdf]

| | **Low risk (n = 176)** | **Medium risk (n = 349)** | **High risk (n = 178)** | **p value** |
|---|---|---|---|---|
| Age (mean)(SD)(years) | 29.68 ±5.68 | 54.92 ±12.01 | 75.29 ±9.93 | **<0.001** |
| Sex<br>Female<br>Male | <br>42 (23.9%)<br>134 (76.1%) | <br>131 (37.5%)<br>218 (62.5) | <br>69 (38.8)<br>109 (61.2) | <br>0.003 |
| Body mass index (mean)(SD)(kg/m$^2$) | 27.31 ±5.07 | 27.36 ±4.52 | 26.17 ±4.33 | 0.013 |
| ASA<br>I<br>II<br>III<br>IV | <br>152 (86.4%)<br>18 (10.2%)<br>6 (3.4%)<br>0 (0%) | <br>163 (46.7%)<br>144 (41.3%)<br>39 (11.2%)<br>3 (0.9) | <br>23 (12.9%)<br>75 (42.1%)<br>68 (38.2%)<br>12 (6.7%) | <br><0.001 |
| Co-morbidity<br>Arterial hypertension<br>Diabetes mellitus<br>Chronic obstructive pulmonary disease<br>Chronic kidney disease<br>Cardiovascular disease<br>Liver disease<br>Active oncological disease | <br>8 (4.5%)<br>1 (0.6%)<br>0 (0%)<br>2 (1.1%)<br>1 (0.6%)<br>1 (0.6%)<br>0 (0%) | <br>94 (26.9%)<br>40 (11.5%)<br>10 (2.9%)<br>8 (2.3%)<br>17 (4.9%)<br>4 (1.1%)<br>7 (2.0%) | <br>120 (67.4%)<br>49 (27.5%)<br>29 (16.3%)<br>12 (6.7%)<br>36 (20.2%)<br>1 (0.6%)<br>7 (3.9%) | <br><0.001<br><0.001<br><0.001<br>0.005<br><0.001<br>0.704<br>0.030 |
| Anticoagulant therapy | 2 (1.1%) | 6 (1.7%) | 14 (7.9%) | <0.001 |
| Antiplatelet therapy | 2 (1.1%) | 9 (2.6%) | 13 (7.3%) | 0.003 |
| Surgical history<br>Umbilical scar<br>Infraumbilical scar | <br>3 (1.7%)<br>54 (30.7%) | <br>20 (5.7%)<br>139 (39.8%) | <br>19 (10.7%)<br>80 (44.9%) | <br>0.002<br>0.020 |
| Charlson comorbidity index (mean) (points) | 0.13 ±0.52 | 1.56 ±1.54 | 4.07 ±1.91 | <0.001 |
| Previous hospitalizations due to gallbladder disease (mean)(SD) | 0.06 ±0.23 | 0.09 ±0.29 | 0.09 ±0.29 | 0.321 |
| History of antibiotic use<br>No<br>Yes | <br>150 (85.2%)<br>26 (14.8%) | <br>297 (85.1%)<br>53 (14.9%) | <br>130 (73.4%)<br>47 (26.6%) | <br>0.002 |
| Palpable mass in right hypochondrium<br>No<br>Yes | <br>173 (98.3%)<br>3 (1.7%) | <br>343 (98.3%)<br>6 (1.7%) | <br>174 (98.3%)<br>3 (1.7) | <br>1.000 |
| Pre-operative laboratories (mean)(SD)<br>Leukocytes (× 10$^3$)<br>Hemoglobin (mg/dL)<br>Bilirubin (mg/dL)<br>Alkaline phosphatase (mg/dL)<br>Aspartate aminotransferase (mg/dL) | <br>10,975.58 ±3,978.74<br>14.55 ±1.65<br>0.98 ±0.95<br>126.36 ±96.80<br>93.83 ±143.87 | <br>10,516.53 ±4,461.92<br>14.69 ±1.83<br>1.47 ±2.30<br>96.65 ±179.05<br>96.65 ±179.05 | <br>10,566.27 ±4,881.47<br>14.36 ±2.05<br>2.44 ±2.96<br>225.11 ±216.30<br>154.18 ±239.04 | <br>0.519<br>0.153<br><0.001<br><br>0.002 |

| | | | | |
|---|---|---|---|---|
| Alanine aminotransferase (mg/dL) | 118.74 ±202.73 | 110.27 ±189.95 | 165.77 ±234.26 | 0.012 |
| C-reactive protein (mg/dL) | 4.45 ±8.57 | 6.49 ±9.74 | 7.26 ±8.52 | 0.166 |
| Imaging findings | | | | |
| Bile duct diameter (mean)(SD)(mm) | 4.28 ±1.34 | 4.67 ±1.60 | 6.20 ±3.25 | <0.001 |
| Gallbladder wall thickness (mean)(SD)(mm) | 3.88 ±2.04 | 3.85 ±1.71 | 3.84 ±1.56 | 0.982 |
| Scleroatrophic gallbladder | 2 (1.1%) | 3 (0.9%) | 1 (0.6%) | 0.841 |
| Perforation | 1 (0.6%) | 7 (2.0%) | 5 (2.8%) | 0.281 |
| Tokyo severity | | | | |
| Without cholecystitis | | | | <0.001 |
| I | 48 (27.3%) | 74 (21.2%) | 22 (12.4%) | |
| II | 32 (18.2%) | 85 (24.4%) | 37 (20.8%) | |
| III | 3 (1.7%) | 30 (8.6%) | 36 (20.2%) | |
| Pre-operative ERCP | | | | |
| No | 176 (100%) | 316 (90.5%) | 97 (54.5%) | <0.001 |
| Yes | 0 (0%) | 33 (9.5%) | 81 (45.5%) | |
| History of cholecystostomy | | | | |
| No | 174 (98.9%) | 343 (98.3%) | 174 (97.8%) | 0.722 |
| Yes | 2 (1.1%) | 6 (1.7%) | 4 (2.2%) | |
| Intraoperative findings according to Nassar score | | | | 0.001 |
| 1 | 61 (34.7%) | 77 (22.1%) | 34 (19.1%) | |
| 2 | 54 (30.7%) | 121 (34.7%) | 56 (31.5%) | |
| 3 | 40 (22.7%) | 63 (18.1%) | 36 (20.2%) | |
| 4 | 11 (6.2%) | 45 (12.9%) | 27 (15.2%) | |
| 5 | 10 (5.7%) | 43 (12.3%) | 25 (14.0%) | |
| Time from admission to procedure (mean)(SD)(days) | 1.98 ±1.42 | 3.08 ±3.66 | 8.26 ±7.94 | <0.001 |
| Conversion to open | | | | |
| No | 173 (98.9%) | 348 (99.7%) | 173 (97.2%) | 0.036 |
| Yes | 2 (1.1%) | 6 (1.7%) | 5 (2.8%) | |
| Type of cholecystectomy | | | | |
| Total | 171 (97.2%) | 333 (95.4%) | 161 (90.4%) | 0.013 |
| Subtotal | 5 (2.8%) | 16 (4.6%) | 17 (9.6%) | |
| Drain use | | | | |
| No | 171 (97.2%) | 324 (92.8%) | 159 (89.3%) | 0.015 |
| Yes | 5 (2.8%) | 25 (7.2%) | 19 (10.7%) | |
| Surgical time (median)(IQR)(minutes) | 76.46 ±39.49 | 84.04 ±36.45 | 90.33 ±48.62 | 0.007 |
| Hospital stay (median)(IQR)(days) | 1.38 ±4.97 | 1.53 ±5.16 | 2.51 ±6.35 | 0.091 |
| Major complication (Clavien-Dindo ≥3) | | | | |
| No | 176 (100%) | 337 (96.6%) | 170 (95.5%) | |

| | | | | |
|---|---|---|---|---|
| Yes | 0 (0%) | 12 (3.4%) | 8 (4.5%) | 0.025 |
| 30-day mortality | | | | |
| No | 176 (100%) | 346 (99.1%) | 175 (98.3%) | 0.226 |
| Yes | 0 (0%) | 2 (0.6%) | 3 (1.7%) | |
